# Comparative Transcriptomics and Metabolomics Uncover the Molecular Basis of Leaf Rust Resistance in Contrasting *Leymus chinensis* Germplasms

**DOI:** 10.3390/ijms26157042

**Published:** 2025-07-22

**Authors:** Wenxin Gao, Peng Gao, Fenghui Guo, Xiangyang Hou

**Affiliations:** 1 College of Grassland Science, Shanxi Agricultural University, Taigu 030801, China; gaowenxin0629@163.com (W.G.); zyjt_721@163.com (P.G.); 2 Key Laboratory of Model Innovation in Forage Production Efficiency, Ministry of Agriculture and Rural Affairs, Taigu 030801, China; 3 Industrial Crop Research Institute, Shanxi Agricultural University, Fenyang 032200, China; guofhui@163.com

**Keywords:** *Leymus chinensis*, *Puccinia* spp., resistance evaluation, transcriptomics, metabolomics

## Abstract

*Leymus chinensis* (Trin.) Tzvel., a vital native forage grass in northern China for ecological restoration and livestock production, faces severe yield losses and grassland degradation due to rust (*Puccinia* spp.) infection. Current control strategies, reliant on chemical interventions, are limited by evolving resistance risks and environmental concerns, while rust-resistant breeding remains hindered by insufficient molecular insights. To address this, we systematically evaluated rust resistance in 24 *L. chinensis* germplasms from diverse geographic origins, identifying six highly resistant (HR) and five extremely susceptible (ES) genotypes. Integrating transcriptomics and metabolomics, we dissected molecular responses to *Puccinia* infection, focusing on contrasting HR (Lc71) and ES (Lc5) germplasms at 48 h post-inoculation. Transcriptomic analysis revealed 1012 differentially expressed genes (DEGs: 247 upregulated, 765 downregulated), with enrichment in cell wall biosynthesis and photosynthesis pathways but suppression of flavonoid synthesis. Metabolomic profiling identified 287 differentially accumulated metabolites (DAMs: 133 upregulated, 188 downregulated), showing significant downregulation of pterocarpans and flavonoids in HR germplasms, alongside upregulated cutin synthesis-related metabolites. Multi-omics integration uncovered 79 co-enriched pathways, pinpointing critical regulatory networks: (1) In the nucleotide metabolism pathway, genes *Lc5Ns011910*, *Lc1Xm057211*, and *Lc4Xm043884* exhibited negative cor-relations with metabolites Deoxycytidine and Cytosine. (2) In flavonoid biosynthesis, *Lc2Xm054924*, *Lc4Xm044161*, *novel.8850*, *Lc2Ns006303*, and *Lc7Ns021884* were linked to naringenin and naringenin-7-O-glucoside accumulation. These candidate genes likely orchestrate rust resistance mechanisms in *L. chinensis*. Our findings advance the molecular understanding of rust resistance and provide actionable targets for breeding resilient germplasms.

## 1. Introduction

*Leymus chinensis* (Trin.) Tzvel., designated as alkali grass, is a perennial rhizomatous forage species within the genus *Leymus* (Poaceae family) [1]. This species dominates Eurasian steppe ecosystems, with its core distribution spanning northeastern China, Inner Mongolia, Hebei, and Shanxi provinces, where it occupies >50% of temperate grasslands [2,3]. As a premium native forage in China, *L. chinensis* has high biomass yield and nutritional richness, with excellent palatability [4]. Moreover, its extensive root system confers multipronged stress tolerance, including robust cold hardiness, drought resistance, and saline–alkaline adaptation [5], underpinning critical roles in restoring degraded grasslands and combating desertification [6].

However, the ecological functions of *L. chinensis* are under significant threat from rust disease. Rust is a common fungal disease in Poaceae grasslands [7]. Pathogenesis primarily targets leaves, sheaths, and stems [8], causing yield losses, quality deterioration, and plant mortality under severe infection [9,10]. Currently, the control of grass rust diseases mainly relies on agricultural practices such as proper planting density, timely pruning, prompt removal of diseased plant residues, and chemical control using fungicides like triadimefon, propiconazole, and myclobutanil [11,12,13], yet faces three inherent constraints: chemical applications risk for environmental contamination, pathogen resistance evolution, and human/livestock toxicity; cultural methods exhibit limited efficacy and cost-effectiveness; both approaches prove impractical for vast natural grasslands with complex ecosystems [14,15]. Therefore, considering environmental friendliness and safety, actively screening, breeding, and utilizing rust-resistant varieties of *L. chinensis* is one of the most economical and effective measures for controlling rust disease in *L. chinensis* [9,16]. The collection of germplasm resources and evaluation of disease resistance are prerequisites for the screening of disease-resistant germplasm resources and breeding of disease-resistant varieties [17].

Plants deploy a layered immune system against pathogens [18,19]. The primary barrier, pattern-triggered immunity (PTI), is initiated when cell-surface pattern recognition receptors (PRRs) detect pathogen-associated molecular patterns (PAMPs) [20,21,22,23], activating basal defenses including reactive oxygen species (ROS) bursts and cell wall fortification. The secondary tier, effector-triggered immunity (ETI), engages when pathogenic effectors suppress PTI; intracellular nucleotide-binding leucine-rich repeat (NLR) proteins then recognize these effectors [24,25], eliciting amplified responses such as hypersensitive response highly resistant (HR) and systemic acquired resistance (SAR) [26,27,28]. Moreover, PTI and ETI do not operate as fully discrete defense tiers. Emerging evidence indicates that ETI activation requires the involvement of PRRs and their co-receptors, while ETI-derived signaling modulates PTI pathways, thereby amplifying PTI responses [29,30].

Recent omics advancements provide unprecedented resolution for dissecting disease resistance mechanisms [31]. Transcriptomics, exemplified by RNA sequencing (RNA-Seq), interrogates genome-wide expression dynamics via high-throughput sequencing [32], while metabolomics, employing liquid chromatography–mass spectrometry and gas chromatography–mass spectrometry (LC-MS/GC-MS), quantifies global metabolite flux [33]. Integration of these approaches overcomes inherent limitations of single-omics studies, elucidating molecular interactions between genes and metabolites during plant–pathogen responses [34,35,36].

*L. chinensis*, with its high nutritional value and stress resistance, has become an important species for ecological restoration and forage production in the northern grasslands of China [37,38]. However, rust disease reduces yield and causes degradation, severely restricting its production application and ecological function [39,40]. Currently, the breeding of rust-resistant *L. chinensis* faces bottlenecks such as unclear genetic background of germplasm resources, lack of resistance evaluation system, and unclear disease resistance molecular mechanisms [41]. Against this background, based on the previously established *L. chinensis* germplasm resource garden (covering wild germplasms from different geographical sources), this study accurately evaluated the rust resistance indoors, screened out germplasms with significantly differentiated resistance levels, and selected typical HR and ES germplasms to conduct joint analysis of transcriptomics and metabolomics. By dissecting the DEGs, DAMs, and their related pathways at 48 h post-inoculation with rust fungus, potential resistance regulatory genes were mined to provide theoretical support for the molecular breeding of rust-resistant *L. chinensis*.

## 2. Results

### 2.1. Identification and Evaluation of Rust Resistance in 24 Leymus chinensis Germplasms Under Laboratory Conditions

**Figure 1:**
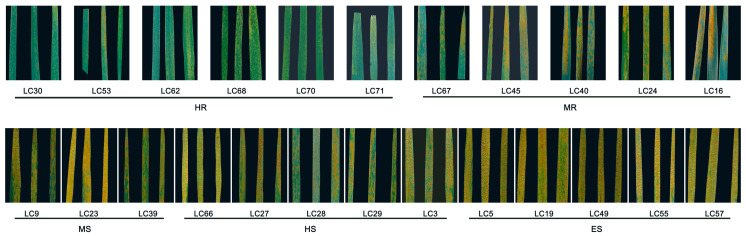
Symptoms of *Leymus chinensis* indoor inoculated with rust after 12 days. Note: Yellowish pustules on leaves represent urediniospore masses of *Puccinia* rust fungus. The percentage of leaf area covered by is uredinia inversely proportional to rust resistance in *L. chinensis* germplasms (greater coverage = weaker resistance).

The resistance evaluation results revealed significant differences in the incidence and disease index (DI) among the 24 *Leymus chinensis* germplasms (*p* < 0.05, Table 1). Disease incidence ranged from 6.67% to 100%, with disease indices spanning 1.33–80.87. Based on DI grading, the germplasms were classified as follows: six highly resistant (HR) (25.00%; Lc30, Lc53, Lc62, Lc68, Lc70, Lc71), five moderately resistant (MR, 20.83%), three moderately susceptible (MS, 12.50%), five highly susceptible (HS, 20.83%), and five extremely susceptible (ES) (20.83%; Lc5, Lc19, Lc49, Lc55, Lc57). No immune germplasms were observed. Representative disease symptoms at 12 days post-inoculation are documented in Figure 1.

The significant differences in rust incidence and DI among these germplasms demonstrate diverse resistance levels. The HR germplasm Lc71 and ES germplasm Lc5 were selected for subsequent molecular analysis.

### 2.2. Transcriptomic Analysis of Resistant and Susceptible L. chinensis in Response to Rust Fungus Infection

#### 2.2.1. RNA Sequencing and Quality Control of Samples

A total of 12 samples from Lc71 (HR) and Lc5 (ES) germplasms—encompassing both inoculated and non-inoculated treatments—underwent reference-based transcriptome sequencing. This yielded 98.79 Gb of Clean Data (high-quality filtered reads), with Q20 (percentage of bases with Phred quality score > 20) and Q30 (quality score >30) and guanine-cytosine (GC) content surpassing 53.42%. These metrics confirm high base-calling accuracy and sequencing integrity, ensuring data reliability for downstream analyses (Table S1).

#### 2.2.2. Analysis of Gene Expression Levels and Principal Component Analysis (PCA) Between Samples

**Figure 2:**
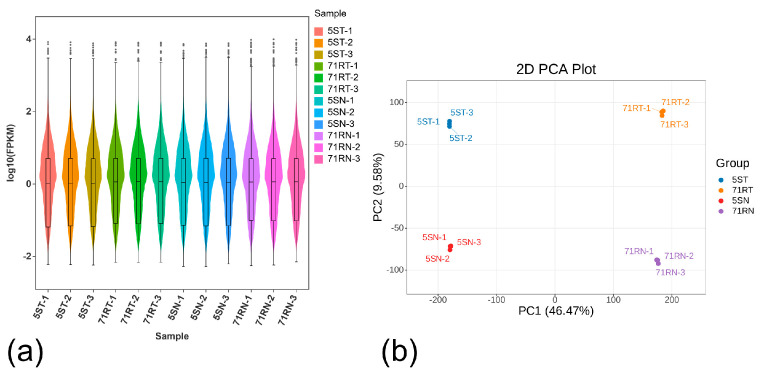
Transcriptome sequencing results and quality assessment: (**a**) Fragments per kilobase per million mapped reads (FPKM) expression violin figure, (**b**) Principal component analysis (PCA) between samples.

Figure 2a shows that the gene expression levels span six orders of magnitude across samples. Although expression distribution and probability density exhibit inter-sample similarity, discernible individual variations persist. Principal component analysis (PCA) of the 12-sample dataset (Figure 2b) reveals significant divergence between treatments of the two germplasms. Crucially, triplicate biological replicates under identical treatments exhibit clustered PC values, confirming minimal intra-group variation alongside substantial inter-group differences, thereby ensuring experimental reproducibility for subsequent analyses.

#### 2.2.3. Screening of Differentially Expressed Genes (DEGs)

The volcano plot of differentially expressed genes DEGs (Figure S1) revealed significant transcriptional reprogramming in resistant and susceptible germplasms following *Puccinia* spp. infection. In the *L. chinensis* Lc5 (ES) treatment group (5ST vs. 5SN), 937 DEGs were identified, comprising 690 upregulated and 247 downregulated genes (Figure S1a). For the *L. chinensis* Lc71 (HR) treatment group (71RT vs. 71RN), 1012 DEGs were detected (247 upregulated and 765 downregulated (Figure S1b), suggesting broader stress-responsive transcriptional regulation in the resistant germplasm. Comparative analysis between genotypes identified 13,600 DEGs in 71RN vs. 5SN (resistant vs. susceptible control; 7201 upregulated), and 14,045 DEGs in 71RT vs. 5ST group (treatment comparison; 7309 upregulated; Figure S1c,d), indicating genotype-specific transcriptional regulation attributable to the resistance phenotype.

**Figure 3:**
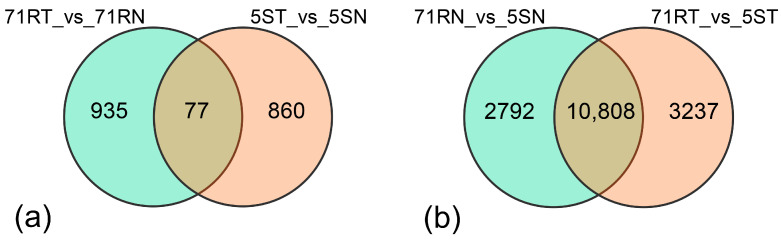
Venn diagram of the differentially expressed genes (DEGs) between R and S: (**a**) Genotype-specific differential genes. (**b**) Treatment-specific differential genes.

Further Venn diagram analysis (Figure 3) elucidated DEG interactions. Seventy-seven core DEGs were shared between *L. chinensis* Lc5 (ES) and *L. chinensis* Lc71 (HR) treatments, with 935 and 860 DEGs exclusive to resistant and susceptible germplasms, respectively (Figure 3a). Cross-group comparisons revealed 10,808 constitutive DEGs shared between 71RN vs. 5SN and 71RT vs. 5ST, alongside 2792 and 3237 treatment-specific DEGs (Figure 3b), demonstrating that resistance formation compromises synergistic actions of genotype-intrinsic differences and stress-induced responses.

#### 2.2.4. Gene Ontology (GO) and Kyoto Encyclopedia of Genes and Genomes (KEGG) Enrichment Analysis of DEGs

**Figure 4:**
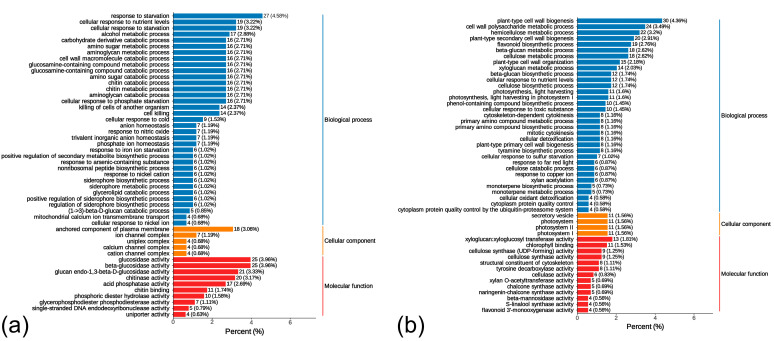
Bar chart of differentially expressed genes (DEGs) gene ontology (GO) enrichment analysis: (**a**) 5ST vs. 5SN, (**b**) 71RT vs. 71RN. Note: The x-axis indicates the number of DEGs, while the y-axis lists GO terms. Labeled numbers denote DEG counts annotated to each term; values in parentheses represent ratios to the total annotated DEGs. Rightmost labels indicate GO categories (BP: biological process, MF: molecular function, CC: cellular component).

Functional analysis of differentially expressed genes (DEGs) based on transcriptomic data revealed distinct gene regulation patterns between *L. chinensis* germplasm Lc71 (HR) and *L. chinensis* Lc5 (ES) germplasms following rust fungus infection. DEGs from the susceptible germplasm *L. chinensis* Lc5 (ES) were significantly enriched in biological processes, including starvation response (GO:0042594, 4.58%) and cell damage (GO:0001906, 2.37%). Upregulation of genes involved in amino sugar catabolism, such as hexosaminidase (*Lc1Xm038572*), could accelerate degradation of cell wall polysaccharides, potentially providing nutritional substrates like N-acetylglucosamine for the pathogen. At the molecular function level, genes associated with β-glucosidase (GO:0008422) and chitinase (GO:0004568) activities constituted 14.42% of DEGs (Figure 4a), and overactivation of cell wall hydrolases resulted in structural integrity loss. Meanwhile, the glycerolipid metabolism pathway exacerbated plasma membrane permeability alterations through membrane lipid hydrolysis, consistent with abnormal enrichment of plasma membrane-anchored component-related genes (GO:0046658) in cellular component analysis (Figure 4a).

DEGs in the *L. chinensis* Lc71 (HR) exhibited systematic defense regulation. In biological processes, genes involved in cell wall biogenesis (GO:0009832, 4.36%) and hemicellulose metabolism (GO:0010410, 3.2%) were significantly activated, and upregulation of cellulose synthase genes (e.g., *Lc4Xm045201*) promoted polysaccharide cross-linking network formation. Molecular function analysis revealed that genes associated with xyloglucan transferase (GO:0016762) and cellulose synthase activity (GO:0016759) constituted 3.06% of DEGs (Figure 4b), directly participating in physical barrier construction. Notably, although flavonoid biosynthesis was generally downregulated in HR, specific upregulation of isoflavone synthase genes (e.g., *Lc2Xm054398*) facilitated antimicrobial compound synthesis via branch-specific metabolic regulation. 

**Figure 5:**
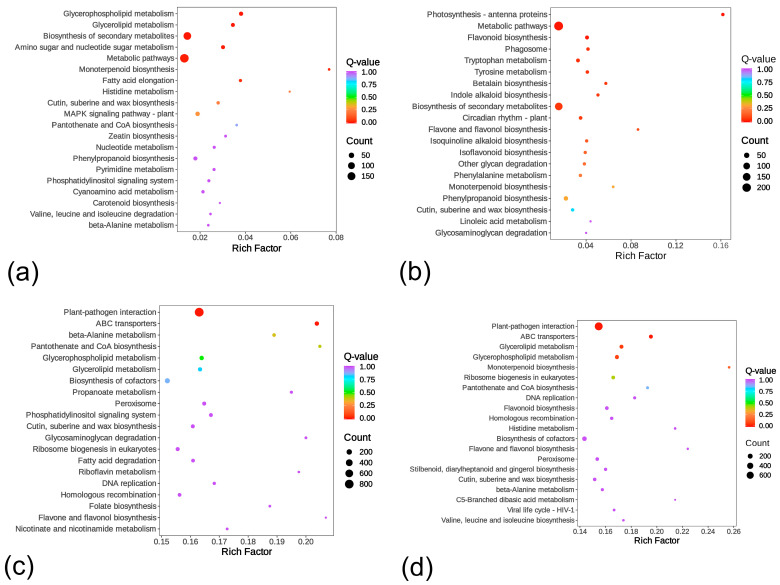
Kyoto encyclopedia of genes and genomes (KEGG) enrichment scatter plot: (**a**) 5ST vs. 5SN, (**b**) 71RT vs. 71RN, (**c**) 71RT vs. 5ST, (**d**) 71RN vs. 5SN. Note: The x-axis indicates the Rich factor (higher values denote greater enrichment). The y-axis lists KEGG pathways. Dot size scales with the number of enriched differentially expressed genes (DEGs) per pathway; color intensity reflects enrichment significance (redder hues indicate higher significance).

Kyoto encyclopedia of genes and genomes (KEGG) pathway analysis further revealed the molecular basis of resistance differentiation (Figure 5). While both germplasms showed significant enrichment in “metabolic pathways” and “biosynthesis of secondary metabolites,” expression patterns were antagonistic. In Lc71 (HR), DEGs were predominantly downregulated, potentially reducing metabolic consumption by suppressing secondary metabolism-related genes (e.g., flavonoids). Conversely, upregulated genes dominated in Lc5 (ES), where abnormal activation of monoterpenoid biosynthesis (ko00902) compromised metabolic homeostasis. This polarity indicates resource reallocation through competitive pathway suppression in HR, whereas metabolic disorder in ES exacerbated coactivation of defense genes and terpenoid synthesis. Germplasm-specific pathway analysis demonstrated that upregulated genes in Lc5 (ES)’s unique amino sugar/nucleotide sugar metabolism facilitated pathogen carbon acquisition, while Lc71 (HR) enriched pathways like plant circadian rhythm (ko04712) and isoflavonoid biosynthesis (ko00943) to coordinate defense gene timing and antimicrobial synthesis.

Cross-group comparisons revealed common enrichment of “plant-pathogen interaction” (ko04626) and “ABC (ATP-binding cassette) transporters” in 71RT vs. 5ST and 71RN vs. 5SN. Differential expression of resistance genes (e.g., TNL (Toll/interleukin-1 receptor-like nucleotide-binding leucine-rich repeat) family) in HR mediated pathogen recognition and hypersensitive response, whereas ABC transporter activation harbored roles in toxin efflux/defense molecule transport. Additionally, significant enrichment of suberin and cutin biosynthesis pathway in 71RT vs. 5ST, coupled with upregulated cell wall reinforcement genes, constituted a physical barrier defense layer. These results demonstrate that Lc71 (HR) achieves multi-level resistance through spatiotemporally transcriptional reprogramming—integrating cell wall fortification, defense compound synthesis, and pathogen recognition signaling. Conversely, metabolic pathway disorder in Lc5 (ES) exacerbated susceptibility.

### 2.3. Metabolomic Reprogramming Signatures in Rust-Infected L. chinensis Germplasms

#### 2.3.1. Metabolomic Data and Quality Control (QC) of Samples 

**Figure 6:**
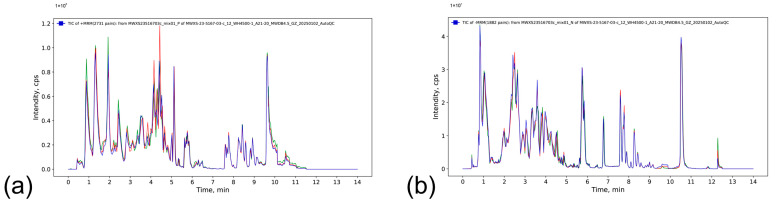
Overlaid total ion current (TIC) chromatograms from quality control (QC) samples: (**a**) P Positive ion mode (P); (**b**) Negative ion mode (N), demonstrating high reproducibility of metabolite detection.

Quality control (QC) samples were prepared from pooled sample extracts. By analyzing the mass spectrometry results of different QC samples (total ion current, TIC, chromatograms), the reproducibility of metabolite extraction and detection can be assessed. As shown in Figure 6, TIC chromatograms exhibited high overlap with consistent retention times and peak intensities, indicating high signal stability across repeated mass spectrometer injections of identical samples.

#### 2.3.2. Principal Component Analysis (PCA) of Metabolites and Validation of Orthogonal Partial Least Squares-Discriminant Analysis (OPLS-DA) Model

**Figure 7:**
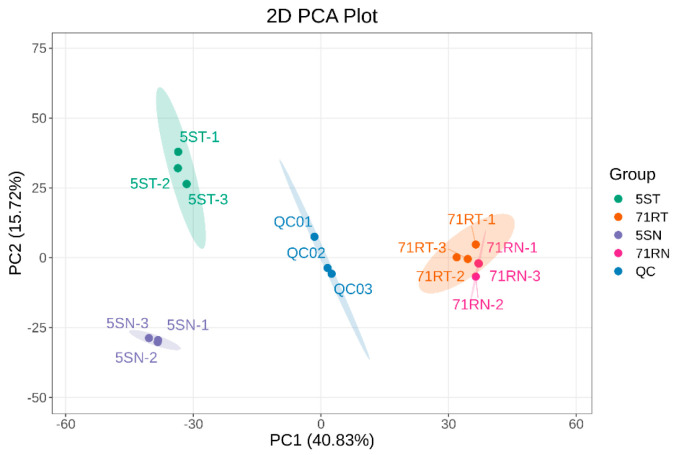
Principal component analysis (PCA) diagram of *Leymus chinensis* sample grouping.

Principal component analysis (PCA) revealed distinct clustering among the 12 experimental samples. Quality control (QC) samples (n = 3) exhibited significant differences in metabolite composition (Figure 7). The first two principal components (PCs) cumulatively captured 56.55% of metabolic variation (PC1 = 40.83%, PC2 = 15.72%). *L. chinensis* Lc71 (HR) and *L. chinensis* Lc5 (ES) germplasms were completely separated along PC1 (Lc71 right, Lc5 left), indicating genotype-driven metabolite profile variation. Treatment vs. control groups were significantly differentiated along the PC2 axis (the treatment group was concentrated in the upper quadrant, while the control group was distributed in the lower quadrant), revealing that the metabolic reprogramming induced by rust fungus infection had commonalities between genotypes. The tight clustering of QC samples in the central area confirms the experimental reproducibility (relative standard deviation, RSD < 15%).

Orthogonal partial least squares-discriminant analysis (OPLS-DA) model validation showed (Table 2) demonstrated optimal explanatory power and predictive ability across all four comparison models: R^2^X = 0.602–0.729, indicating capture of 60.2–72.9% of metabolite variation; R^2^Y = 1.000, confirming completely resolution of inter-group differences; Q^2^ > 0.90 (range 0.913–0.952), affirming excellent predictive performance. These results met the validity threshold of Q^2^ > 0.50 in metabolomics studies and far exceeded the excellent standard of Q^2^ > 0.90, providing a reliable statistical basis for the subsequent screening of differentially accumulated metabolites (DAMs).

#### 2.3.3. Screening of Differentially Accumulated Metabolites (DAMs)

**Figure 8:**
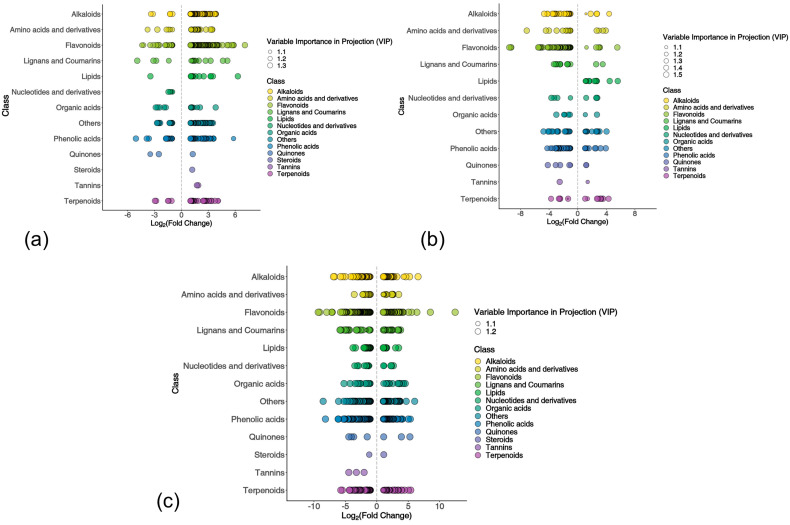
Difference metabolite scatter plot: (**a**) 5ST vs. 5SN, (**b**) 71RT vs. 71RN, (**c**) 71RT vs. 5ST. Note: Each point represents a metabolite (color-coded by class). The x-axis indicates log_2_ (fold change, FC) (group abundance difference, larger absolute values denote greater differential abundance). Point size corresponds to variable importance in projection (VIP) value.

**Figure 9:**
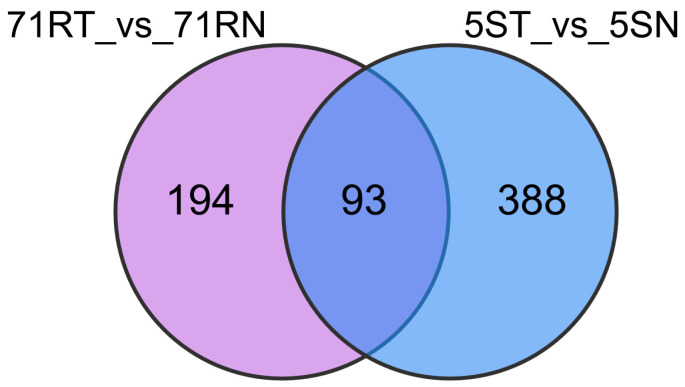
Venn diagram of differential metabolites between resistant (R) and susceptible (S) *Leymus chinensis* germplasms.

Ultra-performance liquid chromatography–tandem mass spectrometry (UPLC-MS/MS) identified 2702 metabolites, revealing significant metabolic reprogramming in *L. chinensis* Lc71 (HR) and susceptible *L. chinensis* Lc5 following rust infection (Figure 8 and Figure 9). Intragroup comparisons showed the Lc5 (ES) treatment group (5ST vs. 5SN) contained 481 DAMs, with 393 (81.7%) significantly upregulated—primarily flavonoids (e.g., Lmgp005868), lipids (PD0677518), and lignans (Lamp007512)—while defensive substances such as phenolic acids (Zmhn002750) and coumarins (Zjgp122320) were downregulated (Figure 8a). In Lc71 (HR) treatment group (71RT vs. 71RN), 287 DAMs included 216 downregulated (75.3%) flavonoids (MWSHY0121) and amino acid derivatives (Lcsp001322), with upregulated alkaloids (Lcsp002898) and terpenoids (Zbzp003763) (Figure 8b), suggesting that it may save resources by suppressing basic metabolism and prioritize the synthesis of specific antimicrobial components.

Inter-group comparisons showed that in the non-inoculated control group (71RN vs. 5SN), there were 962 differential metabolites, with the Lc71 (HR) constitutively accumulating high levels of flavonoids (PD0209862 and others) and phenolic acids (Rlp03049); in the inoculated treatment group (71RT vs. 5ST), there were 865 differential metabolites, with the resistant germplasm specifically upregulating flavonoids and alkaloids, while downregulating competitive metabolites (Figure 8a). Venn analysis further revealed that Lc71 (HR) had 194 unique metabolites, including flavonoid derivatives such as 3,5-trimethoxyflavone glucoside, alkaloids such as caffeoyl-methyl-glucoside, and terpenoids such as apiol (Figure 9). These metabolites play key roles in plant defense, such as tricin 7-O-[feruloyl]-glucoside, which can inhibit the activity of rust fungal extracellular enzymes and hinder infection, and cis-p-coumaroyltyramine, which acts as a phytoalexin precursor involved in the hypersensitive response. In contrast, among the 388 unique metabolites of Lc5 (ES), fatty acid elongation products and monoterpenoids accounted for a significant proportion, and their abnormal accumulation may disrupt cell membrane homeostasis and interfere with normal defense metabolic flux.

The differences in metabolic profiles indicate that the HR optimizes defense by precisely regulating the secondary metabolic network: it suppresses the synthesis of broad-spectrum flavonoids to concentrate resources on the production of highly active antimicrobial compounds (such as nevadensin 7-rutinoside), while specifically accumulating cutin monomers (such as 1-O-feruloylquinic acid) to strengthen the physical barrier. In contrast, the metabolic disorder in ES leads to an imbalance in the synthesis and catabolism of defensive substances, ultimately creating a favorable environment for pathogen proliferation.

#### 2.3.4. Kyoto Encyclopedia of Genes and Genomes (KEGG) Functional Annotation and Enrichment Analysis of Differentially Accumulated Metabolites (DAMs)

**Figure 10:**
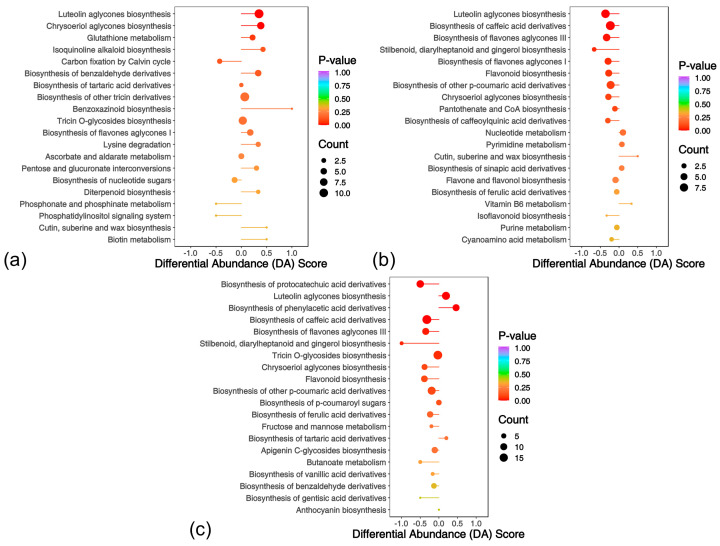
Differential abundance scores of metabolites across treatment combinations: (**a**) 5ST vs. 5SN, (**b**) 71RT vs. 71RN, (**c**) 71RT vs. 5ST.

KEGG pathway enrichment analysis of DAMs revealed fundamentally distinct metabolic regulation strategies in resistant and susceptible *L. chinensis* germplasms post-rust infection (Figure 10). The ES (5ST vs. 5SN) upregulated the biosynthesis of luteolin aglycones, chrysoeriol aglycones, isoquinoline alkaloids, benzaldehyde derivatives, and glutathione metabolism, activating the synthesis of flavonoid antioxidants and enhancing glutathione peroxidase activity to alleviate oxidative damage (Figure 10a). The RH (71RT vs. 71RN) achieved precise defense through metabolic network reprogramming: it downregulated the pathways of stilbenoid, diarylheptanoid and gingerol biosynthesis, flavonoid biosynthesis, luteolin aglycones biosynthesis, and biosynthesis of p-coumaric acid derivatives, inhibiting the synthesis of broad-spectrum antimicrobial substances; meanwhile, it upregulated the pathways of cutin, suberine and wax biosynthesis, nucleotide metabolism, and pyrimidine metabolism (Figure 10b).

In the comparison group of 71RT vs. 5ST (Figure 10c), DAMs were enriched and upregulated in the pathways of luteolin aglycones biosynthesis and biosynthesis of phenylacetic acid derivatives, while they were enriched and downregulated in the pathways of biosynthesis of protocatechuic acid derivatives, stilbenoid, diarylheptanoid and gingerol biosynthesis, chrysoeriol aglycones biosynthesis, flavonoid biosynthesis, biosynthesis of flavones aglycones III, biosynthesis of caffeic acid derivatives, biosynthesis of p-coumaric acid derivatives, and biosynthesis of ferulic acid derivatives.

Overall, the susceptible germplasm attempted to resist the pathogen by upregulating flavonoid compounds and glutathione metabolism, activating basic defense metabolism and the antioxidant system. In contrast, the resistant germplasm downregulated the pathways of stilbenoids; flavonoids; p-coumaric/caffeic acid derivatives; and biosynthesis of pantothenate and CoA, while upregulating the pathways of cutin, suberine, and wax biosynthesis, and nucleotide and pyrimidine metabolism. It is speculated that it resists the pathogen through resource optimization strategies, strengthening physical barriers, and inhibiting pathogen nutrient acquisition.

### 2.4. Coordinated Transcriptomic Metabolomic Dynamics During Rust Infection

#### 2.4.1. KEGG Pathway Co-Enrichment Analysis

**Figure 11:**
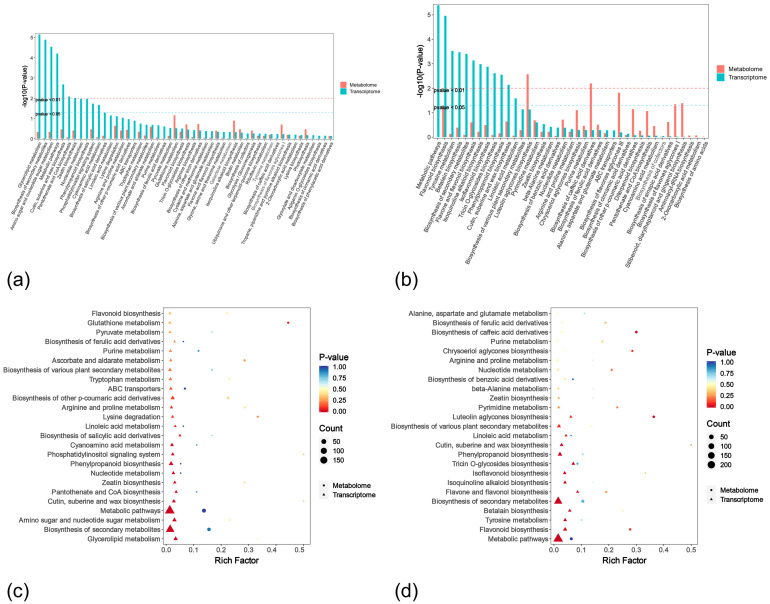
Kyoto encyclopedia of genes and genomes (KEGG) enrichment of differentially expressed genes (DEGs) and differentially accumulated metabolites (DAMs): (**a**) Bar chart for 5ST vs. 5SN; (**b**) Bar chart for 71RT vs. 71RN; (**c**) Bubble chart for 5ST vs. 5SN; (**d**) Bubble chart for 71RT vs. 71RN.

Co-enrichment analysis of differentially expressed genes (DEGs) and DAMs revealed interactive networks in response to rust infection (Figure 11). In the 71RT vs. 71RN comparison group, DEGs and DAMs were co-enriched in 39 KEGG pathways, among which the luteolin biosynthesis and flavonoid biosynthesis metabolic pathways showed relatively higher enrichment of both DAMs and DEGs compared to other pathways. In the flavonoid biosynthesis pathway, DEGs were extremely significantly enriched (*p* < 0.01), while DAMs were enriched but not significantly (*p* > 0.05).

The Lc71 (HR) had unique enriched pathways including flavonoid and flavonol biosynthesis, isoflavonoid biosynthesis, betaine biosynthesis, tyrosine biosynthesis, isoquinoline alkaloid biosynthesis, chrysoeriol aglycones biosynthesis, and cofactor biosynthesis pathways. The Lc5 (ES) had unique enriched pathways in phosphatidylinositol signaling system, lysine degradation, zeatin biosynthesis, linoleic acid metabolism, and glutathione metabolism.

To further elucidate the resistance mechanism, this study selected the flavonoid biosynthesis pathway, which was enriched in both DAMs and DEGs after inoculation in both resistant and susceptible *L. chinensis*, and the nucleotide metabolism pathway, which showed upregulation in DAMs, for in-depth analysis.

#### 2.4.2. Expression Correlation Analysis

**Figure 12:**
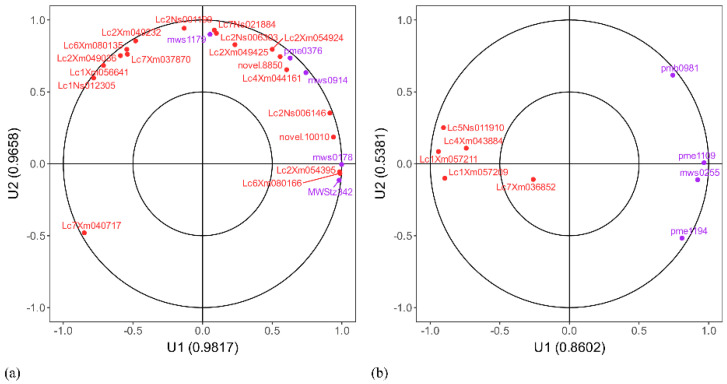
Canonical correlation analysis (CCA): (**a**) ko00941, (**b**) ko01232. Note: Axes show Pearson correlation coefficients between canonical variables (U_1_/U_2_) and metabolites (purple)/genes (red). Distance from origin indicates correlation strength (greater distance = stronger); point proximity reflects similarity in correlation patterns (closer points = more similar).

Canonical correlation analysis (CCA) was employed to elucidate the regulatory network of DEGs and DAMs in the flavonoid biosynthesis (ko00941) and nucleotide metabolism (ko01232) pathways in Lc71 (HR) at 48 h post-inoculation with rust fungus (Figure 11). In the flavonoid pathway (ko00941), genes *Lc2Xm054395* and *Lc6Xm080166* were significantly positively correlated with phenolic acid compounds chlorogenic acid and 5-O-caffeoylquinic acid, with their expression levels being downregulated in concert. Meanwhile, genes *Lc2Xm054924*, *Lc4Xm044161*, *novel.8850*, *Lc2Ns006303*, and *Lc7Ns021884* exhibited significant positive correlations with flavonoids naringenin and naringenin 7-O-beta-D-glucoside (Figure 12a), all showing a downward trend. Notably, gene *Lc7Xm040717* showed a negative correlation with the aforementioned metabolites.

In the nucleotide metabolism pathway (ko01232), genes *Lc5Ns011910*, *Lc1Xm057211*, *Lc4Xm043884*, *mws0255*, and *pme1194* showed negative correlations with the corresponding metabolites, with gene downregulation accompanied by metabolite accumulation. In contrast, genes *Lc1Xm057209* and pmb0981 exhibited negative correlations, characterized by gene upregulation and metabolite downregulation (Figure 12b). These genes play important roles in the flavonoid biosynthesis and nucleotide metabolism pathways, and Lc71 (HR) may respond to pathogen infection by regulating these genes and metabolites in the pathways.

## 3. Discussion

### 3.1. Evaluation of Rust Resistance in Leymus chinensis

*Leymus chinensis*, a native grass species in China, serves not only as an excellent forage but also exhibits multiple stress-resistant traits, including cold resistance, drought tolerance, and saline–alkaline adaptation [42]. As a major disease affecting *L. chinensis*, rust prevalence severely compromises its production and grassland ecological balance [43]. Consequently, evaluating rust resistance in *L. chinensis* germplasm resources and exploring associated resistance genes are critical for improving Poaceae crops and forage grasses [44]. Nevertheless, systematic assessment of rust resistance in Chinese *L. chinensis* germplasm resources remains unavailable. Utilizing 24 germplasms previously collected by our team, this study conducted indoor rust resistance evaluations. The incidence rate (6.67–100%) and disease index (DI) (1.33–80.87) revealed significant resistance variation among germplasms from different geographical origins, consistent with findings by Zhang [9], Miedaner [45], and Liu [46]. Six highly resistant (HR) germplasms were identified: Lc30, Lc53, Lc62, Lc68, Lc70, and Lc71. We posit that their resistance is predominantly attributable to genetic factors, suggesting these accessions may harbor broad-spectrum resistance genes and serve as core breeding materials.

### 3.2. Transcriptomic Analysis of Resistant and Susceptible L. chinensis in Response to Rust Infection

Zeng et al. [47] analyzed transcriptomes of a Tibetan hulless barley germplasm with high powdery mildew resistance. Compared to controls, differentially expressed genes (DEGs) in resistant germplasm were enriched in cell wall synthesis, secondary metabolite biosynthesis, signal transduction, and photosynthesis. Similarly, Bilgin et al. [48] observed frequent downregulation of photosynthesis-related genes across eight plant species under 22 biotic stresses, suggesting an adaptive defense strategy via resource reallocation. Saha et al. [49] mapped rust resistance genes in lentil germplasm using molecular markers. They observed that upon inoculation, resistant germplasm rapidly upregulated cell wall-associated genes (e.g., peroxidase, PRX; peroxidase precursor, PER) and photosynthesis-related genes, whereas susceptible germplasm exhibited enhanced expression of fatty acid metabolism genes. Yan et al. [50] applied mesophyll single-cell analysis to rust-resistant and susceptible maize inbred germplasms. Their results demonstrated that at 48 hours post-inoculation (hpi), susceptible germplasm primarily reprogrammed fatty acid metabolism and monoterpene biosynthesis to combat rust stress, while resistant germplasm maintained homeostasis through cell wall thickening and photosynthetic regulation.

Consistent with these findings, our study revealed that following rust fungus infection, Lc5 (extremely susceptible, ES) responded primarily by regulating cellular components and primary metabolic processes, such as fatty acid metabolism and monoterpene biosynthesis. Lc71 (HR), however, regulated the biosynthesis of metabolites, cell wall synthesis, and photosynthesis-related processes to sustain essential physiological functions and combat rust fungus stress. These results indicate that distinct germplasms employ different strategies against rust infection, with the resistant germplasm placing greater emphasis on sustaining overall plant health status and defense capabilities.

### 3.3. Metabolomic Analysis of Resistant and Susceptible L. chinensis in Response to Rust Infection

Metabolomic analysis revealed significant divergence in metabolic regulation between rust-resistant germplasm Lc71 (HR) and Lc5 (ES) following rust fungus infection. Flavonoid compounds (flavones, isoflavones, etc.) typically function in stress responses by (1) scavenging reactive oxygen species (ROS) and neutralizing free radicals [51]; (2) synthesizing antimicrobial phytoalexins such as luteolin [52]; or (3) interacting with transcription factors to activate immune genes [53,54]. Notably, Huang et al. [55] demonstrated that moderate flavonoid reduction activates alternative defense mechanisms, enhancing Gossypium hirsutum resistance to Fusarium wilt. Similarly, Zuo Tao et al. [56] suppressed catechin synthesis via antisense dihydroflavonol reductase (DFR) expression in poplar, confirming that controlled flavonoid depletion can improve resistance against specific pathogens. Nucleotide metabolism pathways play diverse roles in plant disease resistance, as they can act as signaling molecules directly involved in disease resistance, provide energy and synthetic precursors to support resistance responses, regulate gene expression, and interact with other metabolic pathways such as plant hormone metabolism to collectively enhance plant resistance [57,58,59]. In this study, the Lc5 (ES) attempted to resist the pathogen by upregulating flavonoid compounds and glutathione metabolism, activating the basic defense metabolism and antioxidant system to prevent pathogen infection. Lc71 (HR), on the other hand, downregulated the pathways of stilbenoids, flavonoids, p-coumaric/caffeic acid derivatives, and biosynthesis of pantothenate and CoA, while upregulating nucleotide and pyrimidine metabolism, cutin, suberine, and wax biosynthesis. These findings align with conserved defense signatures reported by Mashabela et al. [60] in Poaceae. Specifically, Lc71 demonstrated rapid mobilization of phenylpropanoid-flavonoid metabolism (e.g., luteolin-7-O-glucuronide accumulation), whereas susceptible Lc5 exhibited dominant fatty acid/terpenoid synthesis. This metabolic bifurcation suggests that preferential phenylpropanoid activation may represent a cross-species resistance indicator in monocot-*Puccinia* pathosystems, warranting validation across diverse host–pathogen combinations.

### 3.4. Integrated Transcriptomic and Metabolomic Analysis of Resistant and Susceptible L. chinensis in Response to Rust Infection

Zhang et al. [58] reported that during late-stage rust infection in rice 48–120 hpi, activation of the jasmonic acid/systemic acquired resistance (JA/SAR) pathway triggered significant enrichment in the phenylpropanoid-lignin biosynthesis pathway, nucleotide sugar metabolism, and tryptophan metabolism. This response led to host cell wall thickening and the formation of continuous lignified rings. However, no significant alterations in fatty acid or monoterpenoid metabolism were detected. Integrated multi-omics analysis demonstrated that the resistant germplasm Lc71 (HR) establishes a multi-tiered defense network via gene–metabolite coregulation. Within the flavonoid biosynthesis pathway (ko00941), the hydroxylase gene *Lc2Xm054395* and the 4-coumarate-CoA ligase gene *Lc6Xm080166* exhibited significant positive correlations with chlorogenic acid and 5-O-caffeoylquinic acid, both being downregulated. This coordinated suppression likely reduces phenolic acid accumulation through inhibition of phenylpropane metabolism, consequently enhancing cell wall lignification. Simultaneously, glycosyltransferase gene *Lc7Xm040717* showed a negative correlation with naringenin glycosides; its upregulation may redirect metabolic precursors toward alternative defense branches via glycosylation modifications.

Cross-group comparisons (71RT vs. 5ST) revealed 79 co-enriched pathways. In the nucleotide metabolism pathway (ko01232), genes *Lc5Ns011910*, *Lc1Xm057211*, *Lc4Xm043884*, and UTP analog *mws0255* displayed negative correlations with metabolite *pme1194*, characterized by concurrent gene downregulation and metabolite accumulation. Conversely, genes *Lc1Xm057209* and *pmb0981* demonstrated inverse regulation patterns with gene upregulation coupled to metabolite depletion. These interactions suggest Lc71 (HR) modulates pathogen response through precise pathway regulation.

Beyond these core pathways, differentially expressed genes (DEGs) in Lc71 (HR) were significantly enriched in flavonoid/flavonol biosynthesis, isoflavonoid biosynthesis, betaine biosynthesis, tyrosine biosynthesis, and isoquinoline alkaloid biosynthesis. Corresponding differentially accumulated metabolites (DAMs) preferentially accumulated in chrysoeriol aglycone biosynthesis and cofactor biosynthesis pathways. In contrast, susceptible Lc5 (ES)-specific DEGs enriched phosphatidylinositol signaling, lysine degradation, zeatin biosynthesis, linoleic acid metabolism, and amino sugar metabolism pathways, with its DAMs dominating glutathione metabolism. Critical genes and metabolites within these pathways constitute pivotal components of disease resistance mechanisms, offering molecular targets for rust-resistant *L. chinensis* breeding.

## 4. Materials and Methods

### 4.1. Materials

The tested rust fungus Ycgx-2 was isolated from rust-infected *Leymus chinensis* leaves collected at the experimental base in Youyu County, Shanxi Province. This pathogen was provided and preserved by the College of Grassland Science, Shanxi Agricultural University.

The 24 assessed *L. chinensis* germplasms (Table 3), originating from heterogeneous habitats across northern China and Mongolia, underwent standardized cultivation using a polyvinyl chloride (PVC) pipe-based system. Germplasms with differential rust resistance were selected based on prior field phenotypic evaluations. In 2022, tiller propagation from identical mother plants facilitated the transplantation of these germplasms into a climate-controlled greenhouse. The specific methodologies are as follows:

A sterilized substrate mixture of garden soil and vermiculite (1:1, *v*/*v*) was prepared through high-pressure moist heat sterilization (121 °C for 25 min, repeated twice). After cooling, the mixture was sealed and stored under sterile conditions. Healthy plants underwent standardized processing: aerial parts were excised 3 cm aboveground using sterile scissors, while rhizomes were stripped and segmented into 5–10 cm sections containing ≥2 stem nodes. Cultivation employed 15 × 15 × 15 cm pots following this protocol: a sterile moist towel was layered at the pot base; sterilized substrate filled two-thirds of the volume; rhizome segments were inserted at original cultivation depth with repeated pot-lifting to compact soil and ensure root–soil contact; after planting, Hoagland nutrient solution was applied thoroughly. Post-acclimation, substrate moisture was maintained at 75 ± 5%. Plants were grown in climate chambers under 16/8 h (light/dark) photoperiod at 25 ± 3 °C, with 50 mL Hoagland solution (Qingdao Haibo Biotechnology Co., Ltd., Qingdao, China; Cat: HB8870) was applied thoroughly. After one year, transplantation to 10×10×8 cm square pots were implemented using a completely randomized block design with three biological replicates per germplasm (6–9 plants per pot).

### 4.2. Evaluation of Rust Resistance in 24 Leymus chinensis Germplasms Under Laboratory Conditions

Ten days prior to inoculation, aerial parts of *L. chinensis* were excised 3 cm above ground. Upon reaching the one-leaf-one-heart stage in regenerated foliage, a rust spore suspension (1 × 10^5^ spores·mL^−1^) was prepared following Reference [61]. Using a handheld pressure sprayer, all 24 germplasms were inoculated until uniform mist films formed on leaf surfaces without droplet formation.

Post-inoculation dew chamber conditions implemented a gradient humidity protocol: 20 min atomization/60 min interval (2 cycles), 5 min atomization/60 min interval (4 cycles), and 1 min atomization/120 min interval, maintaining ≥95% relative humidity for 24 h. Plants were then transferred to sterile culture rooms at 25–28 °C, 45–50% RH, and 16/8 h (light/dark) photoperiod. Hoagland nutrient solution (20 mL/pot) was applied on days 3 and 10 post-inoculation, with regular tiller removal. Disease progression was monitored daily, with severity assessed visually on days 7 and 12 based on maximum symptom expression.

Disease severity grading followed a six-level standard adapted from (NY/T 1443–2007) [62], incorporating *L. chinensis*-specific rust characteristics: Level 0 (no disease), Level 1 (0–5% leaf area covered by urediniospores), Level 2 (5–25%), Level 3 (25–50%), Level 4 (50–75%), and Level 5 (75–100%).

The formula for calculating the diseased leaf rate is as follows:

(1) $$Diseased\;Leaf\;Rate\;(F)=\frac{Number\;of\;Diseased\;Leaves}{Total\;Number\;of\;Leaves\;Investigated}$$

The formula for calculating the disease index (*DI*) is as follows:

(2) $$DI=\left(\frac{\sum \left(X_{i}\times S_{i}\right)}{\sum \left(X_{i}\times S_{max}\right)}\right)\times 100$$

In Equation (2): *DI* is the disease index; *i* is the disease grade; *X_i_* is the number of units at grade *i*; *S_i_* is the representative value of severity at grade *i*; *S_max_* is the maximum severity value.

The six-tiered classification standard for *L. chinensis* rust resistance (Table 4) was established by integrating the disease resistance grading methodologies of *Festuca arundinacea* [63], *Bletilla striata* [64], and *Poa pratensis* [65], with two-year field dynamic monitoring data. The mean *DI* across both years served as the primary grading benchmark. Genotypes classified as ‘ES’ were determined based on severe symptom expression and high pathogen load, consistent with standard plant pathology practices [66].

### 4.3. Inoculation of Rust Fungi and Sampling in Resistant and Susceptible L. chinensis

The contrasting germplasms Lc71 (HR) and Lc5 (ES) were selected for subsequent analysis. After 20 days of pre-cultivation at 3.00 cm stubble height, seedlings were inoculated at the seedling stage following Section 4.2 protocols: treatment groups received Ycgx-2 urediniospore suspension (0.05% Tween-20), while controls received sterile water (0.05% Tween-20), with three biological replicates per treatment. At 24 hours post-inoculation (hpi), plants were transferred to sterile culture rooms. Leaf samples collected at 48 hpi were pooled equally from three replicates and divided for transcriptome sequencing (0.30 g) and wide-targeted metabolomics (0.60 g) [46,67]. Samples were labeled as 5ST (Lc5 treated), 71RT (Lc71 treated), 5SN (Lc5 control), and 71RN (Lc71 control), flash-frozen in liquid nitrogen, and stored at −80 °C. Successful inoculation was confirmed by typical urediniospore pustules on Lc5 leaves at 7 days post-inoculation (dpi) and significant symptom expansion at 10 dpi (controls remained asymptomatic), after which samples were submitted to Wuhan Metware Biotechnology Co., Ltd. (Wuhan, China) for omics analysis.

### 4.4. Transcriptome Sequencing and Data Analysis

Total RNA was extracted from *L. chinensis* leaves using the cetyltrimethylammonium bromide-PBIOZOL (CTAB-PBIOZOL) combined method (Biozol Reagent, BioFlux Co., Ltd., Hangzhou, China; RNA integrity number, RIN ≥ 7.0), with concentration quantified by Qubit 4.0 Fluorometer (Thermo Fisher Scientific, Waltham, MA, USA) and integrity verified via Qsep400 Bio-Fragment Analyzer. (Bioptic Inc., New Taipei, Taiwan, China). Strand-specific complementary DNA (cDNA) libraries were constructed using the Illumina TruSeq™ Stranded mRNA Kit (Illumina Inc., San Diego, CA, USA): poly(A) + mRNA was enriched by Oligo(dT) beads and fragmented in Mg^2+^ buffer (94 °C, 200–300 bp); double-stranded cDNA (ds-cDNA) was synthesized with SuperScript™ IV Reverse Transcriptase using deoxyuridine triphosphate (dUTP) for strand marking; TruSeq™ adapters were ligated and 250–350 bp inserts selected via 2% agarose gel electrophoresis; final amplification involved 15 polymerase chain reaction (PCR) cycles with Phusion™ High-Fidelity DNA Polymerase (Thermo Fisher Scientific, Waltham, MA, USA).. Qualified libraries (size peak: 280 ± 14 bp, coefficient of variation, CV < 5%) underwent paired-end 150 bp (PE150) sequencing on Illumina NovaSeq™ 6000 (Illumina Inc., San Diego, CA, USA; Q30 ≥ 85%).

Bioinformatics analysis followed Wuhan Metware Biotechnology Co., Ltd.’s standard RNA-seq pipeline: (1) Raw data filtering by fastp v0.23.2 (remove adapters, Q20 < 50%, N > 10%, retain Q30 ≥ 85% reads). (2) Alignment to the *L. chinensis* reference genome [68] (dataset doi:10.6084/m9.figshare.24032238) via HISAT2 v2.2.1 (parameters: --n_base_limit 15 --qualified_quality_phred 20; with alignment rate ≥ 85%). (3) Transcript assembly with StringTie v2.2.6 and novel transcript prediction by Coding Potential Calculator 2 (CPC2) v0.1. (4) Gene expression quantification using featureCounts v2.0.3 (default parameters; FPKM normalization) and differentially expressed genes (DEGs) screening via DESeq2 v1.22.1 (absolute log_2_ fold-change |log_2_FC| ≥ 1, Benjamini–Hochberg false discovery rate FDR < 0.05). (5) Gene Ontology/Kyoto Encyclopedia of Genes and Genomes (GO/KEGG) enrichment with clusterProfiler v4.6.0 (hypergeometric test, FDR < 0.05).

### 4.5. Metabolome Sequencing and Data Analysis

Leaf samples were flash-frozen in liquid nitrogen, lyophilized at −50 °C for 63 h, and homogenized to 200 μm powder using a ball mill (Retsch GmbH, Haan, Germany). Exactly 50 mg powder was extracted with 1.2 mL of pre-chilled 70% methanol containing 250 μg/mL lidocaine (internal standard; Sigma-Aldrich, St. Louis, MO, USA). After vortexing and centrifugation, supernatants were filtered through 0.22 μm membranes (Merck Millipore, Burlington, MA, USA) for ultra-performance liquid chromatography–tandem mass spectrometry (UPLC-MS/MS) analysis. Chromatography employed an Agilent SB-C18 column (2.1 × 100 mm, 1.8 μm; Agilent Technologies, Santa Clara, CA, USA) with mobile phases: A = 0.1% formic acid/water, B = 0.1% formic acid/acetonitrile. The gradient program ran: 5% B (0 min) → 95% B (9 min) → 95% B (10 min) → 5% B (11.1 min) → 5% B (14 min) at 0.35 mL/min, 40 °C. Mass spectrometry used electrospray ionization ± mode switching (ESI ±) (ion spray voltage: ±5500 V/−4500 V; source temperature: 500 °C; curtain gas: 25 psi; nebulizer gas: 50 psi) with multiple reaction monitoring (MRM) transitions monitored on quadrupole-linear ion trap mass spectrometry (QTRAP-MS). Data processing involved: peak area extraction by SCIEX OS 3.0 software (AB Sciex, Framingham, MA, USA); Total Useful Signal normalization; Differential metabolite (DM) screening via orthogonal partial least squares-discriminant analysis (OPLS-DA) (R^2^Y > 0.90, Q^2^ > 0.50; thresholds: VIP > 1.0, |log_2_FC| ≥ 1.0, FDR < 0.05), and pathway analysis through KEGG annotation with hypergeometric test (*p* < 0.05) and impact score filtering (> 0.10).

## 5. Conclusions

This study systematically deciphers the molecular basis of rust resistance in *Leymus chinensis* through integrated transcriptomic and metabolomic profiling of contrasting germplasms. Evaluation of 24 geographically diverse accessions identified six highly resistant (HR) (e.g., Lc71) and five extremely susceptible (ES) (e.g., Lc5) genotypes, revealing that HR germplasms orchestrate resistance by prioritizing resource allocation to structural defense and nucleotide signaling. Key mechanisms include the following: (1) Upregulation of cell wall biosynthesis genes (*Lc2Xm054395*, *Lc6Xm080166*) with concurrent suppression of flavonoid pathways in HR germplasms (1012 differentially expressed genes (DEGs)); (2) Metabolic shift toward cutin synthesis (e.g., *1-O-feruloylquinic acid*) reinforcing physical barriers; and (3) Gene–metabolite coregulation in 79 enriched pathways, notably *Lc2Xm054395*/*chlorogenic acid* negative correlation enhancing lignification and nucleotide metabolism fueling defense. These findings provide molecular targets for breeding. Future work should validate candidates (e.g., *Lc7Xm040717*) via CRISPR-Cas9, dissect cutin-phenylpropanoid crosstalk in field environments, and explore nucleotide signaling in monocot immunity.

## Figures and Tables

**Table 1:** Identification of rust resistance in 24 *Leymus chinensis* germplasms under laboratory conditions.

Number	Material	Incidence Rate	Disease Index	Resistance Level
1	Lc3	54.44 ± 5.09% ^bcdefjh^	36.89 ± 2.52 ^cdef^	HS
2	Lc5	66.21 ± 3.35% ^abcde^	56.36 ± 6.30 ^ab^	ES
3	Lc9	42.65 ± 27.52% ^defjhi^	28.31 ± 16.60 ^defgh^	MS
4	Lc16	33.79 ± 21,53% ^ghij^	10.46 ± 6.99 ^hij^	MR
5	Lc19	88.33 ± 13.64% ^a^	46.61 ± 15.92 ^abcd^	ES
6	Lc23	67.95 ± 6.16% ^abcd^	26.22 ± 13.67 ^efgh^	MS
7	Lc24	51.06 ± 15.44% ^cdefgh^	18.58 ± 6.86 ^fghij^	MR
8	Lc27	75.44 ± 14.06% ^abc^	34.16 ± 12.49 ^cdefg^	HS
9	Lc28	71.22 ± 13.16% ^abcd^	35.25 ± 4.55 ^cdefg^	HS
10	Lc29	68.57 ± 13.25% ^abcd^	39.20 ± 11.05 ^bcde^	HS
11	Lc30	12.45 ± 2.93% ^j^	3.26 ± 1.36 ^j^	HR
12	Lc39	56.60 ± 21.77% ^bcdefg^	25.62 ± 15.52 ^efghi^	MS
13	Lc40	36.66 ± 27.28% ^fghij^	16.22 ± 13.67 ^ghij^	MR
14	Lc45	50.75 ± 20.76% ^cdefgh^	16.31 ± 7.87 ^ghij^	MR
15	Lc49	87.43 ± 10.96% ^a^	62.96 ± 16.63 ^a^	ES
16	Lc53	34.68 ± 24.96% ^ghij^	8.15 ± 6.62 ^ij^	HR
17	Lc55	71.19 ± 10.84% ^abcd^	49.95 ± 9.49 ^abc^	ES
18	Lc57	82.10 ± 10.00% ^ab^	60.78 ± 16.90 ^a^	ES
19	Lc62	21.48 ± 11.18% ^ij^	4.30 ± 2.24 ^j^	HR
20	Lc66	64.16 ± 2.57% ^abcdef^	36.16 ± 6.20 ^cdef^	HS
21	Lc67	37.96 ± 15.80%	11.30 ± 5.56 ^hij^	MR
22	Lc68	17.05 ± 7.95% ^ij^	3.41 ± 1.59 ^j^	HR
23	Lc70	12.26 ± 2.15% ^j^	2.45 ± 0.43 ^j^	HR
24	Lc71	26.39 ± 10.49% ^hij^	7.22 ± 2.55 ^ij^	HR

Note: The data in the table are mean values ± standard error; different lowercase letters in the same column indicate significant differences according to Duncan’s new multiple range test (*p* < 0.05).

**Table 2:** Parameters of orthogonal partial least squares-discriminant analysis (OPLS-DA) models.

Group	R^2^X (cum)	R^2^Y (cum)	Q^2^ (cum)
5ST vs. 5SN	0.674	1.000	0.937
71RT vs. 71RN	0.602	1.000	0.904
71RT vs. 5ST	0.723	1.000	0.977
71RN vs. 5SN	0.729	1.000	0.985

**Table 3:** Evaluation of rust-resistant *Leymus chinensis* materials under indoor conditions.

Material	Longitude (°E)	Latitude (°N)	Elevation (m)
Lc3	117°36′	48°44′	81
Lc5	118°41′	49°47′	152
Lc9	113°29′	48°35′	613
Lc16	117°49′	43°17′	830
Lc19	124°14′	47°33′	934
Lc23	118°37′	44°51′	1016
Lc24	117°41′	44°41′	1019
Lc27	106°45′	47°40′	1043
Lc28	106°35′	46°54′	1045
Lc29	113°17′	40°49′	1047
Lc30	107°48′	47°38′	1050
Lc39	115°37′	43°38′	1170
Lc40	113°35′	41°39′	1195
Lc45	110°19′	40°53′	1262
Lc49	113°15′	44°34′	1349
Lc53	115°42′	42°33′	1445
Lc55	103°50′	46°39′	1504
Lc57	106°21′	48°29′	1541
Lc62	102°47′	47°50′	1668
Lc66	113°11′	35°31′	1701
Lc67	113°22′	37°13′	1706
Lc70	118°57′	36°52′	2000
Lc71	116°24′	39°54′	1100

**Table 4:** Grading standards for resistance to rust disease in *Leymus chinensis*.

Level	Disease Index	Resistance Level
1	*DI* = 0	Immunity (I)
2	0 < *DI* ≤ 10	Highly Resistant (HR)
3	10 < *DI* ≤ 20	Moderately Resistant (MR)
4	20 < *DI* ≤ 30	Moderately Susceptible (MS)
5	30 < *DI* ≤ 45	Highly Susceptible (HS)
6	*DI* > 45	Extremely Susceptible (ES)

## Data Availability

The *Leymus chinensis* reference genome dataset is available on Figshare (DOI: 10.6084/m9.figshare.24184921, version 3 of 13 November 2023). Raw transcriptome sequencing data generated in this study are not yet publicly available due to ongoing related research but are accessible from the corresponding author (Xiangyang Hou, houxiangyang@caas.cn) upon reasonable request.

## Supplementary Materials

The following supporting information can be downloaded at: https://www.mdpi.com/article/10.3390/ijms26157042/s1.
