# Supplementary material for: Comparative Transcriptomics and Metabolomics Uncover the Molecular Basis of Leaf Rust Resistance in Contrasting Leymus chinensis Germplasms
Source: Int J Mol Sci. 2025 Jul 22;26(15):7042. doi: 10.3390/ijms26157042 (PMC12346849; doi:10.3390/ijms26157042)
Supplement: Supplementary file 1 [file ijms-26-07042-s001.zip › ijms-3717852-supplementary.pdf]

Table S1. RNA sequencing output statistics

Sample name	Raw read	Filter read	Base number	Error rate	Q20 (%)	Q30 (%)	GC content 3
5SN-1	58516460	56142760	8.42	0.01	98.33	95.20	54.11
5SN-2	58957822	57013488	8.55	0.01	98.35	95.27	53.79
5SN-3	65180066	62440734	9.37	0.01	98.37	95.32	53.65
5ST-1	57426754	53369624	8.01	0.01	98.47	95.60	53.46
5ST-2	60541988	57570328	8.64	0.01	98.40	95.41	53.59
5ST-3	54300148	51553048	7.73	0.01	98.31	95.18	54.35
71RN-1	56291780	53696266	8.05	0.01	98.33	95.20	53.92
71RN-2	59283230	57105876	8.57	0.01	98.34	95.26	53.72
71RN-3	50027266	47840384	7.18	0.01	98.35	95.28	53.70
71RT-1	57183594	54608026	8.19	0.01	98.33	95.22	53.84
71RT-2	53184738	51079894	7.66	0.01	98.09	94.52	53.42
71RT-3	58774342	56155208	8.42	0.01	98.40	95.42	53.50

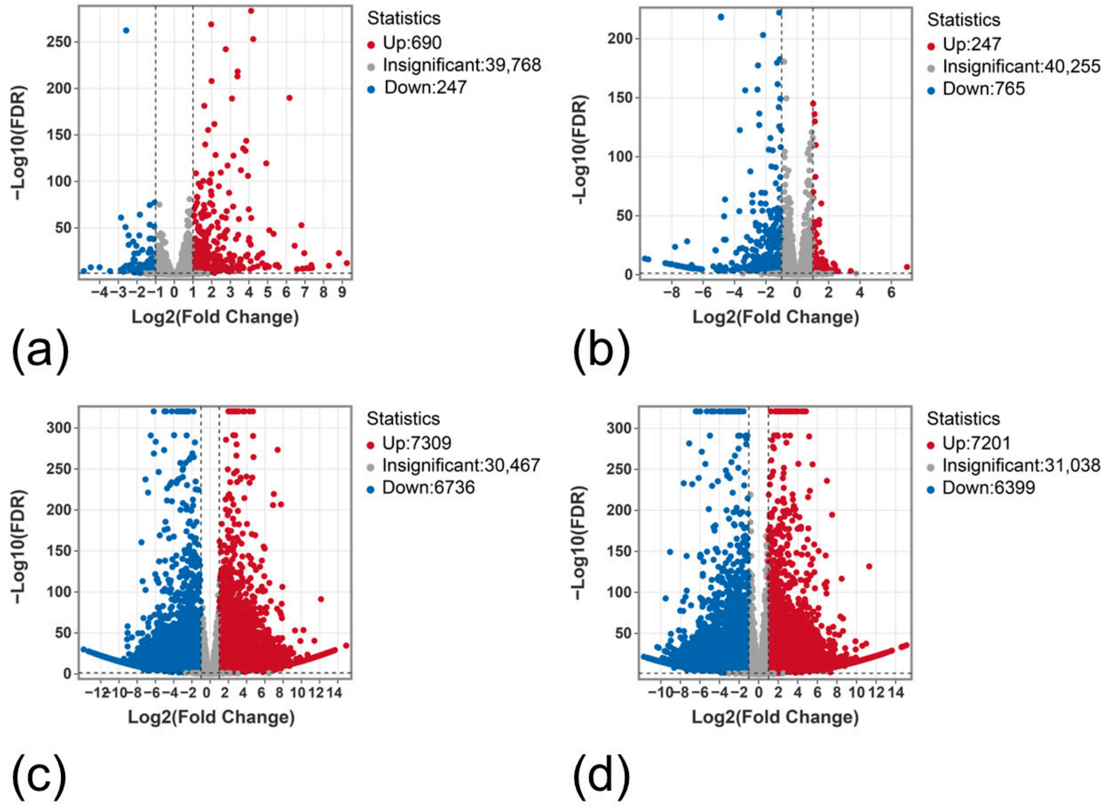

Figure S1. Differential expression gene volcano plot. (a) 5ST vs 5SN (b) 71RT vs 71RN (c) 71RT vs 5ST (d) 71RN vs 5SN. Note: The x-axis represents the fold change in gene expression, and the y-axis represents the significance level of DEGs. Red dots represent upregulated DEGs, blue dots represent downregulated DEGs, and gray dots represent non-DEGs.
